# Targeting Tyro3, Axl, and MerTK Receptor Tyrosine Kinases Significantly Sensitizes Triple-Negative Breast Cancer to CDK4/6 Inhibition

**DOI:** 10.3390/cancers16122253

**Published:** 2024-06-18

**Authors:** Seyma Demirsoy, Ha Tran, Joseph Liu, Yunzhan Li, Shengyu Yang, Dawit Aregawi, Michael J. Glantz, Naduparambil K. Jacob, Vonn Walter, Todd D. Schell, Inan Olmez

**Affiliations:** 1 Departments of Neurosurgery, Penn State University, Hershey, PA 17033, USAmglantz@pennstatehealth.psu.edu (M.J.G.); 2 Department of Radiation Oncology, Ohio State University, Columbus, OH 43210, USA; 3 Departments of Cellular and Molecular Physiology, Penn State University, Hershey, PA 17033, USA; 4 Departments of Public Health Sciences, Penn State University, Hershey, PA 17033, USA; 5 Departments of Microbiology and Immunology, Penn State University, Hershey, PA 17033, USA

**Keywords:** CDK4/6, Axl, MerTK, Tyro3, triple-negative breast cancer, sitravatinib, abemaciclib

## Abstract

**Simple Summary:**

We identified the TAM (Tyro3, Axl, and MerTK) RTKs as a crucial therapeutic vulnerability in Triple-negative breast cancer (TNBC). We show that targeting the TAM receptors with a novel inhibitor, sitravatinib, significantly sensitizes TNBC to CDK4/6 inhibitors. Given the roles of the TAM receptors in promoting the creation of an immunosuppressive tumor microenvironment (TME), we further demonstrate that the combination of CDK4/6 inhibitor abemaciclib and sitravatinib modifies the immune landscape of TNBC to favor immune checkpoint blockade.

**Abstract:**

Triple-negative breast cancer (TNBC) is the most aggressive subtype with high metastasis and mortality rates. Given the lack of actionable targets such as ER and HER2, TNBC still remains an unmet therapeutic challenge. Despite harboring high CDK4/6 expression levels, the efficacy of CDK4/6 inhibition in TNBC has been limited due to the emergence of resistance. The resistance to CDK4/6 inhibition is mainly mediated by *RB1* inactivation. Since our aim is to overcome resistance to CDK4/6 inhibition, in this study, we primarily used the cell lines that do not express *RB1*. Following a screening for activated receptor tyrosine kinases (RTKs) upon CDK4/6 inhibition, we identified the TAM (Tyro3, Axl, and MerTK) RTKs as a crucial therapeutic vulnerability in TNBC. We show that targeting the TAM receptors with a novel inhibitor, sitravatinib, significantly sensitizes TNBC to CDK4/6 inhibitors. Upon prolonged HER2 inhibitor treatment, HER2+ breast cancers suppress HER2 expression, physiologically transforming into TNBC-like cells. We further show that the combined treatment is highly effective against drug-resistant HER2+ breast cancer as well. Following quantitative proteomics and RNA-seq data analysis, we extended our study into the immunophenotyping of TNBC. Given the roles of the TAM receptors in promoting the creation of an immunosuppressive tumor microenvironment (TME), we further demonstrate that the combination of CDK4/6 inhibitor abemaciclib and sitravatinib modifies the immune landscape of TNBC to favor immune checkpoint blockade. Overall, our study offers a novel and highly effective combination therapy against TNBC and potentially treatment-resistant HER2+ breast cancer that can be rapidly moved to the clinic.

## 1. Introduction

Biomarker-based breast cancer classification has led to significant advancement in the treatment of breast cancer patients [1,2]. Target-specific therapeutic agents such as human epidermal growth factor receptor 2 (HER2)-targeting trastuzumab or lapatinib and estrogen receptor (ER)-targeting tamoxifen have been successfully used in the clinic [3,4,5]. Among the breast cancer subtypes, triple-negative breast cancer (TNBC), which accounts for 20% of all breast cancer cases, is the most aggressive subtype with high metastasis and mortality rates [6]. Given the lack of actionable targets such as ER and HER2, therapeutic options against TNBC have remained limited to non-specific chemotherapy and immune checkpoint inhibition [7]. TNBC is highly immunogenic and is associated with high levels of tumor-infiltrating lymphocytes and PD-L1 expression [8,9]. While immune checkpoint inhibitors have shown promising initial results in a subgroup of metastatic TNBC [10,11], due to tumor heterogeneity and the intrinsic resistant nature, the overall efficacy of the current treatments has been unsatisfactory [7,12,13]. Therefore, TNBC still remains an unmet therapeutic challenge and it is highly critical to develop novel targeted therapies with improved efficacy.

CDK4/6 inhibitors such as abemaciclib and palbociclib have become a crucial component of the therapy against early or metastatic ER+/HER2− breast cancers [14,15,16,17]. Subsequently, several preclinical and clinical studies have emerged to test CDK4/6 inhibitors as monotherapy and in combination therapies against TNBC [18]. While TNBC is characterized by significant upregulation of CDK6 expression vs. non-TNBC, the efficacy of CDK4/6 inhibition in TNBC has been limited due to the emergence of resistance.

The TAM (Tyro3, Axl, and MerTK) family of receptor tyrosine kinases (RTKs) has recently been implicated in tumor growth, metastasis, and therapeutic resistance in breast cancer [19,20,21,22,23]. Given their high expression in immune cells, the TAM receptors also play crucial roles in promoting immune evasion and the creation of an immunosuppressive tumor microenvironment (TME) [24,25,26,27], thereby constituting an attractive therapeutic target. With this report, we demonstrate that the TAM receptors are a crucial therapeutic vulnerability in TNBC and treatment-resistant HER2+ breast cancer, the most important therapeutic challenges in breast cancer. We show that targeting the TAM receptors with a novel inhibitor, sitravatinib, significantly sensitizes these tumors to CDK4/6 inhibitors. Recent studies suggest that CDK4/6 inhibition stimulates the anti-tumor immune response [28,29]. Thus, we further demonstrate that the combined CDK4/6 and TAM receptor inhibition significantly activates immune response in TNBC. Overall, our study offers a novel and highly effective combination therapy against TNBC and treatment-resistant HER2+ breast cancer that can be rapidly moved to the clinic.

## 2. Materials and Methods

### 2.1. Small-Molecule Inhibitors

Abemaciclib (LY2835219, S5716), lapatinib (GW-572016, S2111), palbociclib (S4482), and merestinib (LY2801653, S714) were purchased from SelleckChem, Houston, TX, USA. Sitravatinib was received from Mirati Therapeutics (San Diego, CA, USA). All drugs were dissolved in solvents recommended by the manufacturer for in vitro assays. The formula of 5-SD DMSO (ThermoFisher (Waltham, MA, USA), BP231100), 45% PEG-400 (Rikagu Reagents, Bainbridge Island, MA, USA, 1008415,) and 50% purified water (Invitrogen Life Technologies (Waltham, MA, USA), 10977015) was used for in vivo studies.

### 2.2. Cell Culture, Cell Viability Detection, Reagents, and Clonogenic Assay

All breast cancer cell (BCC) lines were obtained from the American Type Culture Collection (ATCC). BCCs were cultured either in DMEM (Dulbecco’s Modified Eagle Medium) (ThermoFisher (Waltham, MA, USA), 11965118) or in RPMI (ThermoFisher, 11875119), supplemented with 10% FBS (R&D Systems (Minneapolis, MN, USA), S11150H) and 1% penicillin/streptomycin (5000 U/mL, Gibco (Waltham, MA, USA), 1507063). All cell lines were maintained in the incubator at 37 °C and 5% CO_2_ for a maximum of 20 passages and regularly checked for mycoplasma contamination. For drug sensitivity assays, BCCs were seeded onto 96-well plates in quadruplicate, and the next day, drug treatment or vehicle (*v*:*v* DMSO) was initiated. Following three days of treatment, cell viability was measured with trypan blue exclusion/cell counting using Cellometer Auto T4 (Nexcelom, Lawrence, MA, USA) and alamarBlue (ThermoFisher Scientific, Waltham, MA, USA) according to the manufacturer’s instructions. Clonogenic assay was performed as described previously [30]. Recombinant human HGF and Gas6 (R&D Systems) were used for pathway stimulation. To show that HGF-induced c-Met signaling transactivates Axl, BCCs were treated with 40 ng/mL HGF for 15 min. Cells were then lysed for immunoblot.

### 2.3. Immunoblotting and Antibody Array (ELISA)

Immunoblotting was performed as previously described [31]. Proteins were separated using SDS-PAGE (Gel = Bolt 4–12% Bis-Tris, Invitrogen (Waltham, MA, USA), NW04120BOX), transferred onto the PVDF membranes (Immobilon-FL, Millipore Sigma, (St. Louis, MO, USA), IPFL00005), and subsequently blocked (Intercept Blocking Buffer, Li-Cor, Lincoln, NE, USA, 92760001) for 1 h prior to the addition of primary antibodies. The following antibodies were used for immunoblotting: phospho-Met (Tyr1234/1235) (CST, 3077), Met (D1C2) (CST, 8198), Axl (C89E7) (CST, 8661), phospho-Axl (Y779) (R&D Systems, MAB6965), phospho-MerTK (Phosphosolutions, Denver, CO, USA, p186-749), MerTK (Abcam, Cambridge, UK, ab52968), phospho-Akt (CST, 9271), phospho-mTOR (abclonal, AP0094), and ERBB2 (CST, 2165). Actin (A5441) and GAPDH (G9545) were from Sigma-Aldrich (St. Louis, MO, USA). IRDye 800CW Goat anti-Rabbit IgG Secondary Antibody and IRDye 680RD Goat anti-Mouse IgG Secondary Antibody (Li-Cor, 35571, 926-32211 and 926-68070, respectively) were used as the secondary antibodies. Images were taken and quantified using Odyssey (Li-Cor Biosciences, Lincoln, NE, USA). The RTK Phosphorylation Array was purchased from Ray Biotech (AAH-PRTK-G1). The assay was performed 24 h after the treatment with abemaciclib or palbociclib according to the manufacturer’s instructions.

### 2.4. Caspase-3/7 Assay and qRT-PCR

The Caspase-Glo 3/7 Assay kit (G8090, Promega) was used for detecting caspase-3/7 levels according to the manufacturer’s instructions following 2 days of drug treatment. Total RNA was isolated using QIAzol reagent (Qiagen, Hilden, Germany) and reverse transcribed using SuperScript III First Strand kit (Invitrogen). qRT-PCR was performed with 2 µL of diluted cDNA on an Applied Biosystems StepOnePlus PCR machine using Power SYBR Green (Applied Biosystems, Waltham, MA, USA) according to the manufacturer’s instructions. Relative quantification was performed for each sample and normalized to GAPDH expression for comparison. The following primers were used: GAPDH: sense, 5′-GAAGGTGAAGGTCGGAGTCA-3′, and antisense, 5′-TTGAGGTCAATGAAGGGGTC-3′; ERBB2 [32]: sense, 5′-ACACCTAGCGGAGCGATG-3′, and antisense, 5′-CATCCCCTTGGCAATCTG-3′; AXL [32]: sense, 5′-ACACCCCAGAGGTGCTAATG-3′, and antisense, 5′-ACGAGAAGGCAGGAGTTGAA-3′; MERTK [33]: sense, 5′-CTCTGGCGTAGAGCTATCACT-3′, and antisense, 5′-AGGCTGGGTTGGTGAAAACA-3′; and MET [34]: sense, 5′-TGATGATGAGGTGGACACA-3′, and antisense, 5′-CTATGGCAAGGAGCAAAGA-3′.

### 2.5. Mass Spectrometry Sample Preparation

Samples for protein analysis were prepared essentially as previously described [35,36]. Following lysis, protein precipitation, reduction/alkylation, and digestion, peptides were quantified by BCA assay, and 150 µg of peptide per sample was labeled with TMT reagents (Thermo-Fisher) for 2 h at room temperature. Labeling reactions were quenched with 0.5% hydroxylamine and acidified with TFA. Acidified peptides were combined and desalted by Sep-Pak (Waters). Following enrichment, phosphopeptides were desalted via Stage Tips and re-dissolved in 5% formic acid/5% acetonitrile. Peptides from the flow-through were further fractionated for full proteome analysis.

### 2.6. Basic pH Reversed-Phase Separation (BPRP)

TMT-labeled peptides were solubilized in 5% ACN/10 mM ammonium bicarbonate, pH 8.0, and 300 µg of TMT-labeled peptides was separated by an Agilent 300 Extend C18 column (3.5 μm particles, 4.6 mm ID, and 250 mm in length). An Agilent 1260 binary pump coupled with a photodiode array (PDA) detector (Thermo Scientific) was used to separate the peptides. A 45 min linear gradient from 10% to 40% acetonitrile in 10 mM ammonium bicarbonate pH 8.0 (flow rate of 0.6 mL/min) separated the peptide mixtures into a total of 96 fractions (36 s). A total of 96 fractions were consolidated into 24 samples, acidified with 20 µL of 10% formic acid, and vacuum-dried to completion. Each sample was desalted via Stage Tips and re-dissolved in 5% formic acid/5% acetonitrile for LC-MS3 analysis.

### 2.7. Mass Spectrometry Data Collection (LC-MS3)—Total Proteome

Proteome data were collected on an Orbitrap Fusion Lumos mass spectrometer (ThermoFisher Scientific) coupled to a Proxeon EASY-nLC 1000 LC pump (ThermoFisher Scientific). Fractionated peptides were separated using a 150 min gradient at 600 nL/min on a 35 cm column (i.d. 100 μm, Accucore, 2.6 μm, 150 Å) packed in-house. MS1 data were collected in the Orbitrap (120,000 resolution; maximum injection time 50 ms; AGC 10 × 10^5^). Charge states between 2 and 5 were required for MS2 analysis, and a 180 s dynamic exclusion window was used. Top 10 MS2 scans were performed in the ion trap with CID fragmentation (isolation window 0.5 Da; Turbo; normalized collision energy—35%; maximum injection time 50 ms; AGC 2 × 10^4^). An on-line real-time search algorithm (Orbiter) was used to trigger MS3 scans for quantification [37]. MS3 scans were collected in the Orbitrap using a resolution of 50,000, NCE of 55%, maximum injection time of 200 ms, and AGC of 3.0 × 10^5^. The close out was set at two peptides per protein per fraction [37].

### 2.8. Total Proteome Data Analysis

Raw files were converted to mzXML, and monoisotopic peaks were re-assigned using Monocle [38]. Searches were performed using the Comet search algorithm against a mouse database downloaded from Uniprot in February 2014. We used a 50 ppm precursor ion tolerance, 1.0005 fragment ion tolerance, and 0.4 fragment bin offset for MS2 scans collected in the ion trap. TMTpro on lysine residues and peptide N-termini (+304.2071 Da) and carbamidomethylation of cysteine residues (+57.0215 Da) were set as static modifications, while oxidation of methionine residues (+15.9949 Da) was set as a variable modification.

Each run was filtered separately to 1% false discovery rate (FDR) on the peptide–spectrum match (PSM) level. Then, proteins were filtered to the target 1% FDR level across the entire combined data set. Phosphorylation site localization was determined using the AScore algorithm [39]. For reporter ion quantification, a 0.003 Da window around the theoretical m/z of each reporter ion was scanned, and the most intense m/z was used. Reporter ion intensities were adjusted to correct for isotopic impurities of the different TMTpro reagents according to manufacturer specifications. Peptides were filtered to include only those with a summed signal to noise (SN) ≥ 100 across all TMT channels. For each protein, the filtered peptide TMTpro SN values were summed to generate protein or phosphorylation site quantification values. To control for different total protein loading within a TMTpro experiment, the summed protein quantities of each channel were adjusted to be equal within the experiment.

### 2.9. Tissue Microarray (TMA) and IHC Staining

Breast cancer TMA slides were purchased from Biomax (#BR1202a). Immunohistochemistry was performed on a robotic platform (Ventana discover Ultra Staining Module, Ventana Co., Tucson, AZ, USA), as described before [40]. Slides were first fixed with acetone–methanol (1:1 ratio) for 10 min. Endogenous peroxidases were blocked with peroxidase inhibitor (CM1) for 8 min and then incubated with the Met (CST, 8198), MerTK (Abcam, ab52968), and Axl (CST, 8661) antibodies at 1:100 dilution for 60 min at room temperature. Antigen–antibody complexes were then detected using the DISCOVERY OmniMap Anti-Rb HRP detection system and DISCOVERY ChromoMap DAB Kit (Ventana Co.).

### 2.10. Animal Studies

All animal studies were approved by Penn State University Institutional Animal Care and Use Committee (IACUC). SKBR3, SKBR3 LapR, or HCC1806 (500,000 or 1 million) were injected into the 4th mammary fat pad of six-to-eight-week-old female Crl:NU(NCr)-Foxn1nu (Charles River Laboratories) or CBySmn.Cg-Prkdcscid/J (The Jakson Laboratory) mice. For studies with 4T1, 2 million cells were injected into the 4th mammary fat pad of six-to-eight-week-old female BALB/cJ (Jackson Laboratories) mice. Tumor volume was followed by caliper measurements every four days. Once tumor sizes reached 3–5 mm, mice were randomized into 4 groups: control, sitravatinib only, abemaciclib only, and the combined treatment. We followed two types of treatment regimens; in the continuous treatment arm, both sitravatinib (10 mg/kg/day) and abemaciclib (50 mg/kg/day) were given once daily for 6 days a week, and in the alternating treatment, each drug was given to the designated groups alternating two days on, two days combined, and two days off (Abe, Abe, Abe + Sitra, Abe + Sitra, Sitra, Sitra). No animals were excluded from the analysis.

A BCM-2147 patient-derived xenograft (PDX) model was maintained in female Fox Chase SCID Beige (Charles River Laboratories) mice. After the tumor size reached 1000 mm^3^, the mice were sacrificed, and after removal, the tumor was cut into 1 mm fragments. Each tumor fragment was then implanted into the mammary fat pad of female Crl:NU(NCr)-Foxn1nu (Charles River Laboratories) mice, as described previously [41]. Briefly, anesthetized mice were positioned in a supine position. A 5 mm longitudinal incision was made either at the #2 or #4 mammary gland level. Subcutaneous tissue was dissected to expose the mammary fat pad, and a 1 mm³ tumor piece was inserted at the center using fine-point forceps. The midline incision was then sealed with tissue clips. The animals were randomized into different treatment groups when the tumors reached approximately 200 mm^3^. Mice were treated following the alternating regimen.

### 2.11. Immune Profiling and Flow Cytometry

For immune response evaluation, 4T1 cells (2 million per mouse) were injected into the 4th mammary fat pad of six-to-eight-week-old female BALB/cJ (Jackson Laboratories) mice. Once tumor sizes reached 3–5 mm, mice were randomized into 4 groups, as above. Mice were treated for 3 weeks according to the alternating treatment regimen and euthanized with xylazine/ketamine injection followed by cervical dislocation. After tumors were removed from the site, they were minced into small pieces and digested with cocktail containing collagenase II (1 mg/mL, C6885-100MG, Millipore Sigma) and DNaseI (0.1 mg/mL, DN25100MG, Millipore Sigma) at 37 °C for 15 min by mixing occasionally. After incubation, the cells were smashed on a 40 µm strainer using the back of a 1 mL syringe and washed with 10 mL PBS twice and centrifuged at 400× *g* for 5 min. Then, the cells were counted and frozen in 10% DMSO and 90% FBS at a concentration of 5 × 10^6^ cells/mL.

The frozen tumor cells were thawed and counted using trypan blue. A total of 1 × 10^6^ cells/well were transferred into 96-well round-bottom plates. Samples were stained with 1:1000 diluted LIVE/DEAD Fixable Stains (FVS780, Cat # 565388) in PBS for 15 min at room temperature. Following a wash step, cells were resuspended in FcR Blocking Reagent (BD Biosciences, Mouse Fc Block Cat # 553142) at a 1:100 dilution in FACs buffer (2% fetal bovine serum and 0.02% NaN3 in PBS) for 15 min on ice. Fluorophore-conjugated antibodies, diluted 1:50, were added to the suspensions, and cells were further incubated for 15 min at room temperature in the dark. Samples were washed with PBS/BSA and fixed overnight at 4 °C or for 2 h at room temperature in Fixation Buffer. Subsequently, the cells were washed, resuspended in PBS, and transferred into flow microtiter tubes (Fisher Cat # 02-681-376). Samples were acquired with a 17-color or 23-color BD FACS Symphony and analyzed using FlowJo (v10.7.2, BD Biosciences). The following antibodies were used: CD45 (clone 30-F11, 553080, BD Biosciences), CD3 (clone 500A2, 553240), CD4 (RM4-5, 566407, BD Biosciences), CD8 (clone 53–6.7, 566985, BD Biosciences), CD279 (J43, 745546, BD Biosciences), CD11b (M1/70, 552850, BD Biosciences), CD49b (DX5, 563063, BD Biosciences), F4/80 (T45-2342, 752152, BD Biosciences), Ly6G (clone 1A8, 563979, BD Biosciences), Ly6C (clone AL-21, 563011, BD Biosciences), MHC II (clone 2G9, 746669, BD Biosciences), and CD11c (clone N418, 745852, BD Biosciences). One-way ANOVA with Tukey correction was used for the comparisons among the study groups. Specific immune populations were then graphed.

### 2.12. Bioinformatical Analyses

The limma R package 4.3.2 [42] was employed to perform differential expression analysis based on gene-level reads per kilobase per million mapped reads (RPKM) values from RNA sequencing (RNAseq); similar analyses were performed for gene-level protein measurements. Lowly expressed genes were removed from the RNAseq analyses. For each comparison of interest, statistical significance was assessed using a false discovery rate (FDR) threshold of 0.05. Gene set enrichment analysis (GSEA [43,44]) was applied using the pre-ranked approach with both Hallmark and KEGG gene sets [43,45]. For each comparison of interest, genes were ranked according to the *t*-test statistic from the corresponding differential expression analysis. GSEA enrichment scores for select gene sets were compared among cell line treatment groups. All analyses were performed with R 4.2.2 [46].

### 2.13. Statistics and Synergy Calculations

GraphPad Prism 10 (GraphPad Software) was used for statistical analysis. Student’s *t*-test was utilized for 2-group comparisons. For multiple comparisons, both one-way ANOVA with post hoc Tukey analysis and one ANOVA with Dunnett’s multiple comparisons test analysis and one-way ANOVA on ranks with Dunn’s multiple comparison test analysis were utilized. *p*-values less than 0.05 were considered significant using an error rate of α = 0.05. Sample sizes were chosen based on our prior experience and power calculation of 85%. We utilized two types of synergy calculation methods, the Bliss difference and the Chou–Talalay (ComboSyn 1.0). The Bliss difference was calculated as described previously [47]. The Bliss value is found by subtracting the predicted cytotoxicity from the observed cytotoxicity of a combination therapy. When the Bliss value is zero, two individual treatments are considered additive, whereas greater than zero indicates synergy, and less than zero indicates antagonism. This method is informative even when one of the components of a combination therapy fails to produce a notable response. Combination indices (CIs) were generated using the Chou–Talalay method. CI < 1 is considered to be synergistic, and CI < 0.2 is considered strong synergy [48].

## 3. Results

### 3.1. Certain Receptor Tyrosine Kinases (RTKs) Are Hyperactivated in Response to CDK4/6 Inhibition in TNBC

**Figure 1:**
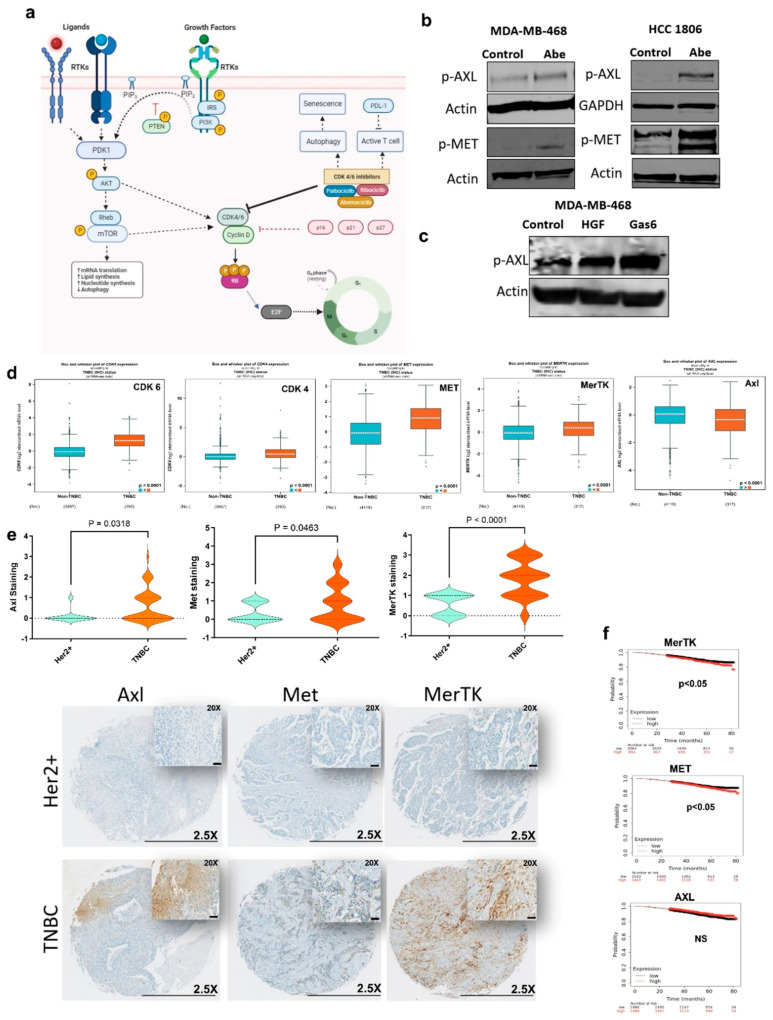
TAM/Met receptor tyrosine kinases are upregulated in TNBC. (**a**) Schematic representation of receptor tyrosine kinase-mediated regulation of CDK4/6. (**b**,**c**) Immunoblot was performed on cell lines treated for 24 h with Abe (2 μM) (**b**) and for 25 min with either HGF (40 ng/mL) or Gas6 (400 ng/mL) (**c**). Protein levels were determined for phospho-AXL and phospho−MET. (**d**) Comparison of gene expression levels in TNBC vs. non-TNBC, based on RNAseq data from breast cancer patients. (**e**) TMA IHC staining for total Axl, Met, and MerTK in TNBC and HER2+ breast cancer (lower panel). Scale bars are 0.5 mm for 2.5× and 50 μm for 20×. Violin plots show the quantification of each protein expression based on the H-scoring in TNBC vs. HER2+ (two-tailed *t*-test). (**f**) The Kaplan–Meier survival estimate for MerTK, Met, and Axl based on the RNAseq data from all breast cancer patients. Abe: abemaciclib. The original western blot figures can be found in File S1.

CDK4/6 activity is regulated by multiple factors including Akt and mTOR that subsequently control the cell cycle (Figure 1a). Using TNBC cell lines, we initially showed that Akt and mTOR are hyperactivated upon treatment with CDK4/6 inhibitors abemaciclib and palbociclib (Supplementary Figure S1a). Akt and mTOR are primarily regulated by the upstream RTKs. RTKs play a critical role in tumor growth, therapeutic resistance, metastasis, and the creation of an immunosuppressive tumor microenvironment. We therefore questioned if certain RTKs are activated in response to CDK4/6 inhibition. Using a phospho-RTK array, we found significant increases in the activities of Met and Axl RTKs compared with the other RTKs following 24 h abemaciclib or palbociclib treatment (Supplementary Figure S1b). We subsequently confirmed the hyperactivation of Met and Axl with immunoblotting following treatment with abemaciclib and palbociclib (Figure 1b and Supplementary Figure S2a). Since Axl is a member of the TAM family of RTKs, we also showed the hyperactivation of another TAM member, MerTK (Supplementary Figure S2a), which was not tested with the phospho-RTK array. The transactivation of RTKs is a well-known adaptive response that results in more potent downstream signaling through the oncogenic mediators [49]. We therefore questioned the presence of crosstalk between Met and Axl RTKs, leading to enhanced resistance to CDK4/6 inhibition. With an HGF stimulation test that includes the brief treatment of TNBC cells with HGF, the ligand for Met, we showed an increase in the activity of Axl (Figure 1c), suggesting the transactivation between Met and Axl.

TNBC is characterized by elevated CDK6 vs. non-TNBC. Using the gene expression database on breast cancer patients [50], we found that besides CDK6, Met and the TAM RTKs other than Axl are significantly upregulated in TNBC vs. non-TNBC (Figure 1d and Supplementary Figure S2b). Using the TCGA database for breast cancer patients (cBioPortal, PanCancer Atlas), we also showed that CDK6 expression significantly correlates with the expression of Met and the TAM RTKs, Axl, MerTK, and Tyro3, and is inversely correlated with HER2 (Supplementary Figure S2c). We further confirmed these differential expression levels using patient tumor microarrays. We showed that the protein expression levels of Met, MerTK, and Axl are significantly higher in TNBC vs. HER2+ patients (Figure 1e). Of note, we found that while the upregulation of Met and MerTK is associated with shortened patient survival, Axl expression has no impact on survival (Figure 1f). Altogether, our findings suggest that the TAM/Met axis could be a crucial therapeutic vulnerability for TNBC.

### 3.2. Simultaneous TAM/Met and CDK4/6 Inhibition Has Synergistic Activity against TNBC

**Figure 2:**
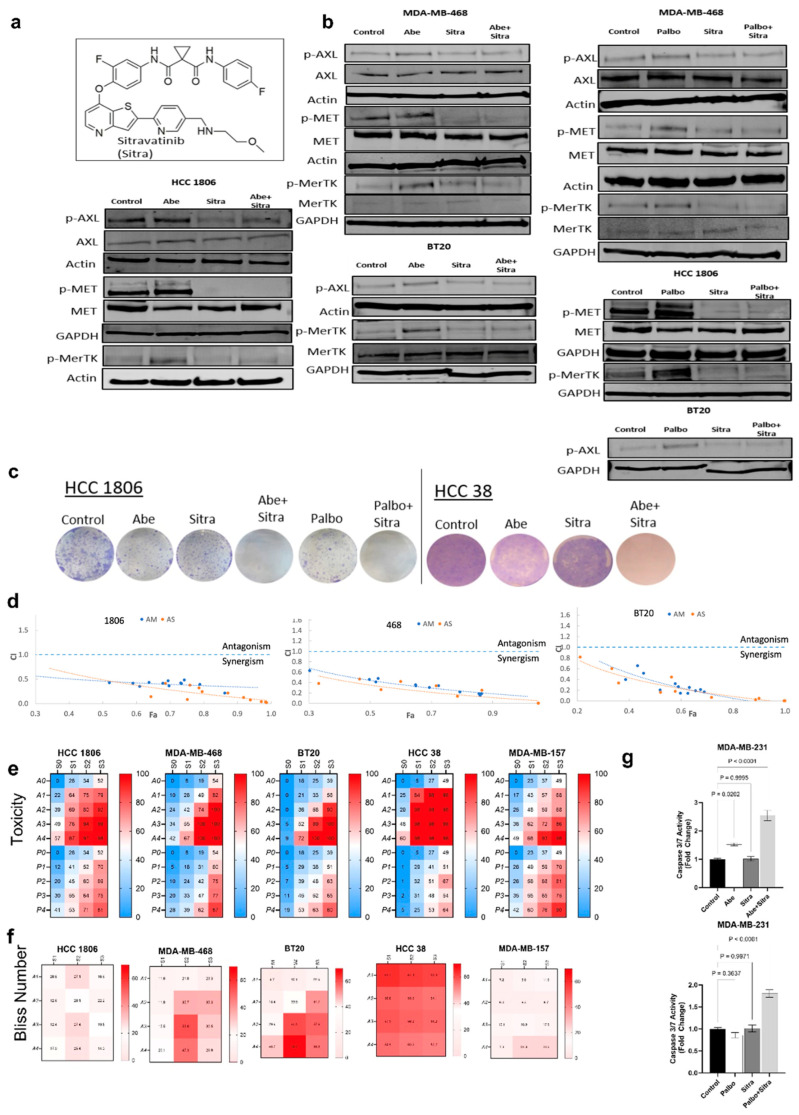
The combination of sitravatinib with abemaciclib or palbociclib is highly toxic against TNBC cells. (**a**) Chemical structure of sitravatinib (Sitra). (**b**) Immunoblot was performed on cell lines treated for 24 h with Abe (2 μm), Palbo (5 μm), and/or Sitra (2 μm). Protein levels were determined for phospho-AXL, phosho-MET, and phosho-MERTK. (**c**) The clonogenic assay showing that the combination of Abe or Palbo with Sitra significantly decreased the colony formation capacity of TNBC cells. Representative images of stained colonies. (**d**) Combination index (CI) values for the combinations of sitravatinib or merestinib with CDK4/6 inhibitor abemaciclib using different doses. Circles represent experimentally determined CI values using the Chou–Talalay method. The colors (orange and blue) represent the fixed ratio mixtures. (**e**,**f**) Overview of the toxicity and synergy scores of the drug combinations for TNBC lines. The heatmaps show the level of toxicity (**e**) and Bliss number (**f**) for the cell lines tested in this study. Average values of toxicity (**e**) or Bliss number (**f**) for cells treated with sitravatinib (S) at varying doses (S0 = No Drug, S1 = 1 μm, S2 = 2 μm, and S3 = 3 μm) in combination with either abemaciclib (A) at varying doses (A0 = No Drug, A1 = 1 μm, A2 =2 μm, A3 = 3 μm, and A4 = 4 μm) or palbociclib at varying doses (P0 = No Drug, P1 = 1 μm, P2 = 2 μm, P3 = 3 μm, and P4 = 4 μm). (**g**) Shown is the caspase-3/7 activity measured upon 24 h of drug treatments. The data are presented as mean ± SEM from three independent experiments, expressed as ratios to untreated control values, with associated *p* values as indicated (One-way ANOVA with Dunnett’s multiple comparisons test analysis). Abe: abemaciclib; Palbo: palbociclib. The original western blot figures can be found in File S1.

We therefore tested a novel TAM/Met inhibitor, sitravatinib, for the first time in combination with two CDK4/6 inhibitors abemaciclib and palbociclib against TNBC. Sitravatinib is a potent and orally bioavailable small-molecule inhibitor (Figure 2a). Sitravatinib was successfully evaluated in phase 1 and phase 2 clinical trials singly and in combination against non-small-cell lung cancer (NSCLC) and renal cancers [51,52,53,54]. The first phase 3 trial of sitravatinib in combination with nivolumab vs. docetaxel for the treatment of NSCLC showed promising results [55]. However, its clinical efficacy against breast cancer has not yet been tested.

We initially demonstrated that, when combined with abemaciclib or palbociclib, sitravatinib significantly reduced the activities of Met, Axl, and MerTK, which were hyperactivated following abemaciclib or palbociclib treatment (Figure 2b), and their downstream mediators Akt and mTOR (Supplementary Figure S3b). We also used another small-molecule inhibitor that primarily targets TAM/Met, merestinib (Supplementary Figure S3a). We showed that merestinib treatment similarly reverses the hyperactivation of Met, Axl, and MerTK upon CDK4/6 inhibition (Supplementary Figure S3b). This suggested that combined TAM/Met inhibition could help overcome the resistance to CDK4/6 inhibitors in TNBC. We subsequently evaluated the efficacy of sitravatinib in combination with abemaciclib or palbociclib against TNBC lines in vitro. With the clonogenic assay, we showed that the combined treatment at low doses significantly suppressed cellular proliferation and colony formation (Figure 2c). We found similar results with the combination of merestinib and CDK4/6 inhibitors (Supplementary Figure S3c). Testing a range of doses, we demonstrated that the combination of sitravatinib and CDK4/6 inhibitors exhibited significant synergy against TNBC cell lines calculated using two different statistical methods (Figure 2d–f). We further confirmed substantial synergy using merestinib in combination with abemaciclib or palbociclib against TNBC lines (Supplementary Figure S3d,e). CDK4/6 inhibitors are cytostatic agents—they typically do not induce apoptosis compared to the cytotoxic agents [56]. Given the enhanced cellular toxicity, we tested whether the combined TAM/Met and CDK4/6 inhibition triggered apoptosis. We showed with a caspase-3/7 activity assay that the combined treatment significantly induced apoptosis, while there was no apoptosis in the individual treatments (Figure 2g and Supplementary Figure S3f).

### 3.3. Combined TAM/Met and CDK4/6 Inhibition Exhibits Enhanced Cytotoxicity against Drug-Resistant HER2+ Breast Cancer

**Figure 3:**
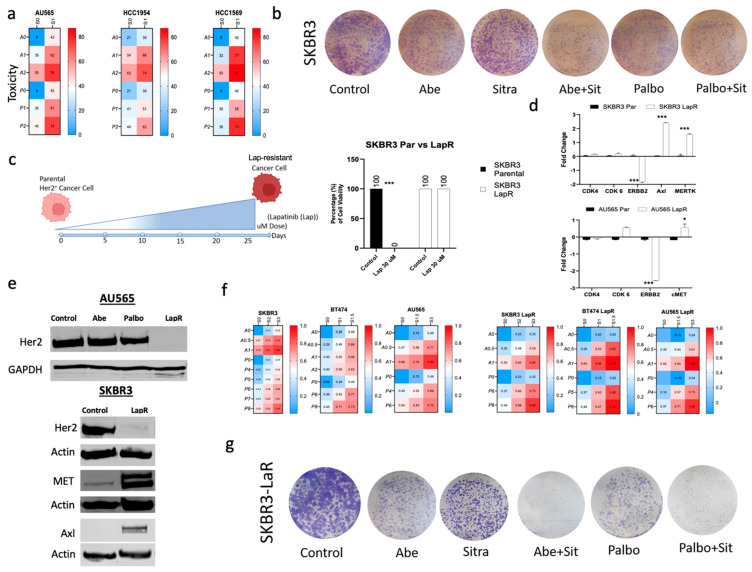
Lapatinib-resistant HER2+ cell lines became more sensitive to the combination of sitravatinib with abemaciclib or palbociclib. (**a**) Overview of the toxicity of the drug combinations for HER2+ cell lines. The heatmaps show the level of toxicity for the cell lines tested. Average values of toxicity for cells treated with sitravatinib (S) at varying doses (S0 = No Drug, S1 = 1 μm) in combination with either abemaciclib (A) (A0 = No Drug, A1 = 1 μm, and A2 = 2 μm) or palbociclib (P0 = No Drug, P1 = 1 μm, and P2 = 2 μm). (**b**) The clonogenic assay showing that the combination of Abe or Palbo with Sitra had only modest effect on the HER2+ cell line SKBR3. Representative images of stained colonies. (**c**) Schematic representation of the generation of lapatinib-resistant (LapR) HER2 lines through continuous lapatinib treatment with gradual increase in treatment dose up to 30 μm. Cell viability confirming the resistance of the LapR cells to high doses of lapatinib (30 μm). (**d**,**e**) qRT-PCR and immunoblot showing increased expressions of Axl, Met, and MerTK with the suppression of Her2 levels in LapR vs. the parental cells. (**f**) Cell viability showing increased sensitivity of SKBR3 LapR cells to the combination of abemaciclib or palbociclib with sitravatinib compared with the parental SKBR3 cells. Overview of the toxicity of the drug combinations for HER2+ and LapR HER2 cell lines. The heatmaps show the level of toxicity for the cell lines tested. Average values of toxicity for cells treated with sitravatinib (S) at varying doses (S0 = No Drug, S1 = 1 μm, and S2 = 2 μm) in combination with either abemaciclib (A) (A0 = No Drug, A1 = 1 μm, A2 = 2 μm, and A3 = 3 μm) or palbociclib (P0 = No Drug, P1 = 1 μm, and P2 = 2 μm). (**g**) The clonogenic assay showing that SKBR3-LapR cells became highly sensitive to the combination of Abe or Palbo with Sitra. Representative images of stained colonies. Abe: abemaciclib; Palbo: palbociclib; Sitra: sitravatinib. Each bar represents mean ± SEM from three independent experiments, with associated *p* (* *p* < 0.05, *** *p* < 0.0001; one-way ANOVA with post hoc Tukey analysis). The original western blot figures can be found in File S1.

Given the lower expression of TAM/Met in HER2+ breast cancer, as expected, we showed that combined TAM/Met and CDK4/6 inhibition exhibited less efficacy in HER2+ breast cancer lines (Figure 3a,b and Supplementary Figure S4a). Since extended treatment with the HER2 inhibitors results in the suppression of HER2 expression, we questioned whether prolonged treatment with an HER2 inhibitor lapatinib sensitizes HER2+ breast cancer lines to combined TAM/Met and CDK4/6 inhibition. We generated lapatinib-resistant HER2+ breast cancer lines through treatment with gradually increasing doses of lapatinib and confirmed the development of resistance even to the very high doses of lapatinib (Figure 3c). Using immunoblot and qRT-PCR, we initially determined that lapatinib-resistant lines significantly suppressed HER2 expression (Figure 3d,e), thus physiologically transforming into TNBC-like cells. We subsequently demonstrated that, similar to TNBC cell lines, lapatinib-resistant cells harbored significantly elevated Met, MerTK, and Axl expressions (Figure 3d,e). We then showed that the upregulation of Met, MerTK, and Axl significantly sensitized lapatinib-resistant cells to combined TAM/Met and CDK4/6 inhibition vs. the parental HER2+ cells (Figure 3f–h and Supplementary Figure S4b,c).

### 3.4. The Combination of TAM/Met and CDK4/6 Inhibition Is Highly Effective against TNBC and Drug-Resistant HER2+ Breast Cancer In Vivo

**Figure 4:**
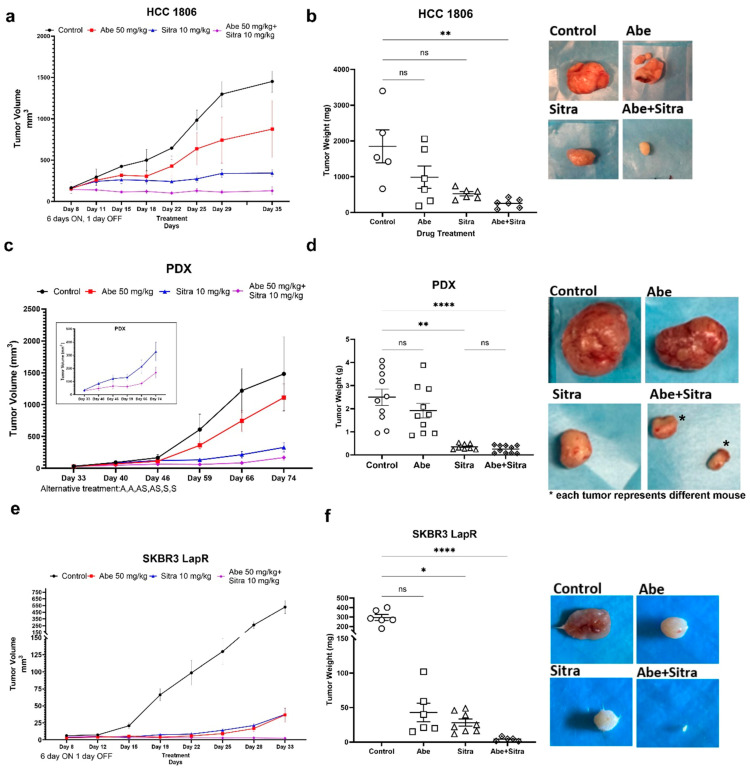
The combined treatment suppresses tumor growth in TNBC models. (**a**,**c**,**e**) The combination of abemaciclib and sitravatinib significantly suppressed tumor growth in the HCC1806 xenograft (**a**) and BCM-2147 patient-derived xenograft (PDX) (**c**) models using Crl:NU(NCr)-Foxn1nu nude mice and in the SKBR3-LapR xenograft model (**e**) using CBySmn.Cg-Prkdcscid/J mice. The treatment schedules, continuous or alternating treatment, are indicated on the graphics. In the continuous treatment arm, both sitravatinib (10 mg/kg/day) and abemaciclib (50 mg/kg/day) were given once daily for 6 days a week, and in the alternating treatment, each drug was given to the designated groups alternating two days on, two days combined, and two days off (Abe, Abe, Abe+Sitra, Abe+Sitra, Sitra, Sitra). No animals were excluded from the analysis. Tumor sizes were detected with caliber measurements and compared across the treatment groups (n = 6/group). (**b**,**d**,**f**) The combined therapy significantly reduced tumor weights compared to the control group with associated *p* (* *p* < 0.05, ** *p* < 0.01, **** *p* < 0.0001); one-way ANOVA on ranks with Dunn’s multiple comparison test analysis). ns: not significant. Shown are the representative tumor images for each treatment.

Next, we evaluated the efficacy of the sitravatinib and abemaciclib combination using two types of treatment regimens. With continuous treatment, mice were treated with vehicle, once-daily oral abemaciclib (50 mg/kg/day, six days a week), once-daily oral sitravatinib (10 mg/kg/day, six days a week), or the combination of abemaciclib and sitravatinib. With the alternating treatment, each drug was given to the designated groups alternating two days on, two days combined, and two days off. The tumor volume was followed by regular caliper measurements, and tumor weights were compared upon study termination. We initially tested the efficacy of the combined treatment in TNBC xenografts, HCC1806 line, and BCM-2147 patient-derived xenograft (PDX) models. We showed that the combined treatment significantly suppressed tumor growth in these models (Figure 4a–d and Supplementary Figure S5a). We next tested the efficacy of the combined treatment in untreated parental and lapatinib-resistant HER2+ breast cancer xenograft models. We found that while there was no significant difference across the treatment groups in the parental HER2+ breast cancer xenograft model (Supplementary Figure S5c), the combined treatment significantly suppressed tumor formation and growth in the drug-resistant HER2+ xenograft model (Figure 4e,f and Supplementary Figure S5b). Notably, there was no difference in the average body weights of mice across the treatment groups, and no overt toxicity was observed in the individual or combined drug treatments (Supplementary Figure S5d). Overall, these results suggest a high therapeutic potential of the combination of sitravatinib and abemaciclib preferentially in TNBC and drug-resistant HER2+ breast cancer.

### 3.5. The Combined Treatment Reverses Immunosuppressive Tumor Microenvironment

**Figure 5:**
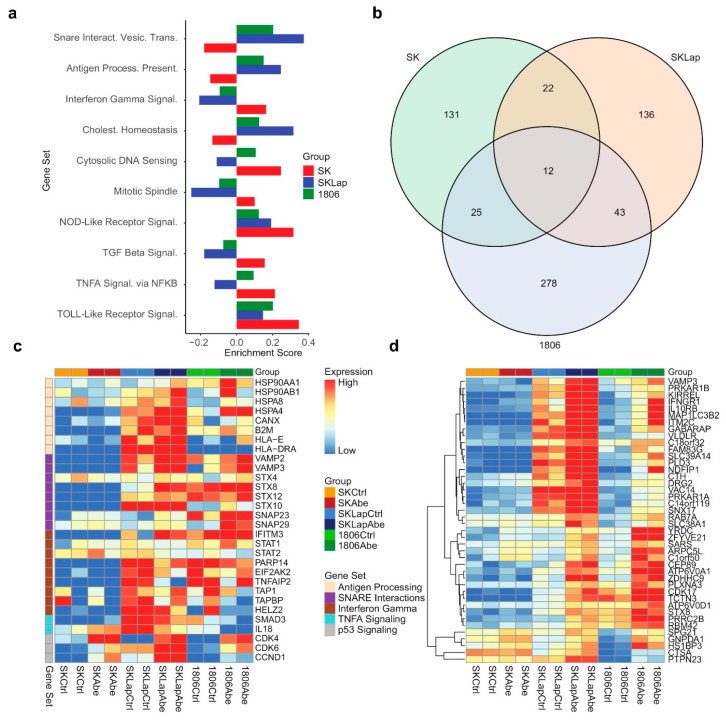
Results from proteomics differential expression analysis. (**a**) Bar plot display of enrichment scores from a gene set enrichment analysis (GSEA) pre-ranked analysis for select Hallmark and KEGG gene sets. (**b**) Venn diagram shows counts of proteins that were upregulated in treatment vs. control for each of the three cell lines based on a false discovery rate threshold of q = 0.15. (**c**,**d**) Proteomics expression heatmaps for proteins of interest, where proteins are labeled by gene. Columns are grouped by cell line and treatment in both panels. Rows are grouped by gene set in panel (**c**); rows are hierarchically clustered in panel (**d**). Expression values are mean-centered by row in each panel. Proteins in panel c were selected from gene sets of interest, as shown in the figure legend; proteins in panel d were upregulated in treatment vs. control for both SKLapR and 1806 cell lines but not SKBR3 (SK), as shown in the Venn diagram below.

We next explored functional changes at the protein level in response to CDK4/6 inhibition that may potentially contribute to the hypersensitivity of TNBC to the combined treatment. For this, we performed differential expression analyses of quantitative proteomics measurements to compare protein abundance levels in TNBC (HCC1806), HER2+ (SKBR3), and lapatinib-resistant HER2+ (SKBR3-LapR) upon abemaciclib treatment for 24 h. Gene set enrichment analysis (GSEA) for Hallmark and KEGG pathway gene sets was performed using the GSEA pre-ranked approach and results of the differential expression analysis, and the enrichment scores were compared. Our analysis showed significant enrichment of proteins associated with cellular communication and the immune response process, including SNARE interactions and antigen presentation in TNBC and lapatinib-resistant HER2+ vs. parental HER2+ cell lines (Figure 5a,c). We supplemented our proteomics investigation with analyses of RNA-seq data (GSE99116) obtained from a recent study where different CDK4/6 inhibitors were compared for transcriptional changes in breast cancer cell lines [57]. Similar to our proteomics findings, the GSEA of the RNA-seq data for Hallmark and KEGG pathway gene sets showed the enrichment of genes associated with SNARE interactions and antigen presentation in TNBC cell lines following 24 h abemaciclib treatment (Supplementary Figure S6). We then identified the proteins that were upregulated in the treatment vs. control for both SKBR3-LapR and 1806 cell lines but not SKBR3 (Figure 5b,d). Using this list of genes, we performed functional enrichment analysis for Gene Ontology (GO) on the g:Profiler platform [58]. We found that several biological processes were activated in HCC1806 and SKBR3-LapR such as autophagy, cell communication, vesicle-mediated transport, and lysosomal activity (Supplementary Figure S7a). Using Western blot, we validated these findings by examining some of the specific markers associated with the endo-lysosomal pathway, crucial for immune response, antigen presentation, signal transduction, and cell communication. Our results revealed an upregulation of LC3 and a concurrent downregulation of p62. Treatment with chloroquine (CQ) indicated that the observed increase may not necessarily be attributed to impaired autophagic activity; instead, it suggested an augmentation in autophagic activity, although further confirmation is warranted. Additionally, we noted a heightened expression of CD81, a protein commonly associated with exosomes and recognized as a classical marker for extracellular vesicles [59] (Supplementary Figure S7b). These observations collectively signify the alterations in the intracellular compartments originating from the endosome/lysosome pathway. Among the top upregulated proteins, we identified the ones that are associated with immune evasion and inflammation (Supplementary Figure S7c). Given the role of the TAM RTKs in the creation of an immunosuppressive TME and the immunostimulant activity of sitravatinib, we subsequently sought to evaluate the impact of the combined treatment on immune response.

**Figure 6:**
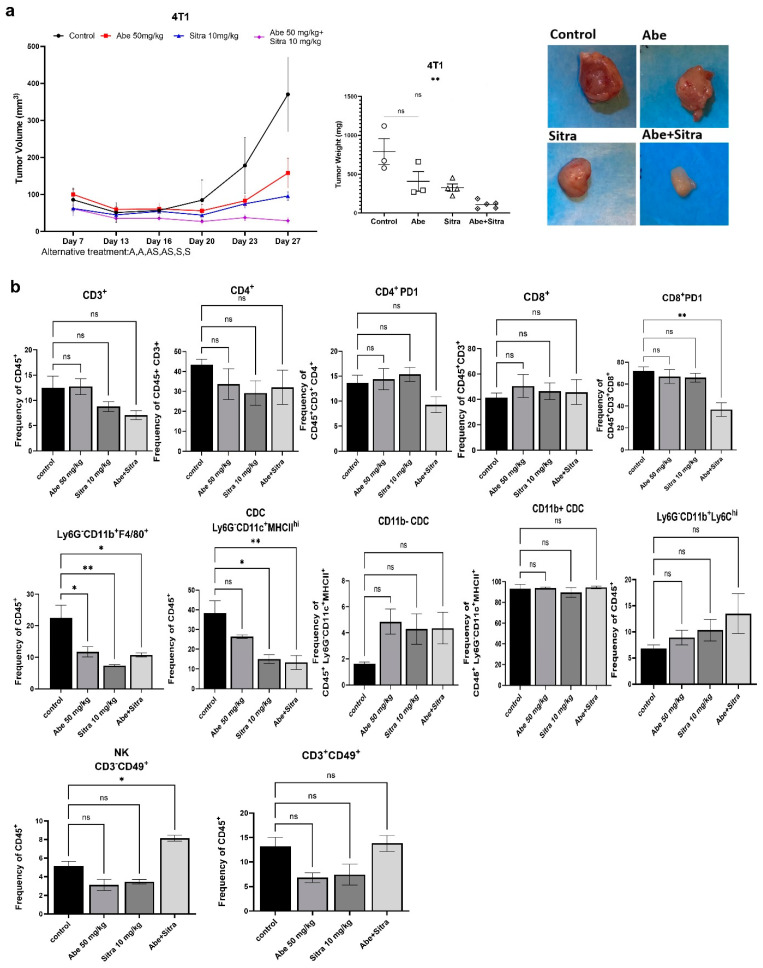
The combined treatment induces anti-tumor immune response. (**a**) The combination of abemaciclib and sitravatinib significantly suppressed tumor growth in immunocompetent Balb/c mice using mouse TNBC line 4T1. Tumor sizes were detected with caliber measurements. Tumor weights were compared across the treatment groups (** *p* < 0.01, one-way ANOVA on ranks with Dunn’s multiple comparison test analysis). ns: not significant. (**b**) Bar plots of the frequency of tumor-infiltrating immune cells out of shown values in 4T1 tumors at day 21 of the treatment, as measured by BD FACS Symphony (*n* = 3 or 4 per treatment group). *p* values were detected using one-way ANOVA with Dunnett’s multiple comparisons test analysis. * *p* < 0.05, ** *p* < 0.01, ns: not significant.

We subcutaneously implanted mouse TNBC 4T1 cells into immunocompetent BALB/c mice. Mice were then randomized into four groups: control, sitravatinib only, abemaciclib only, and the combined treatment. We followed an alternating treatment regimen; each drug was given to the designated groups alternating two days on, two days combined, and two days off. The tumor volume was followed by regular caliper measurements, and tumor weights were compared upon study termination. We demonstrated that the combined treatment significantly suppressed tumor growth (Figure 6a). In a parallel experiment, we examined the impact of the combined treatment on the immune landscape of tumors. After randomization, mice received treatment according to the alternating schedule for 20 days. Tumor-derived single-cell suspensions were stained and analyzed by multicolor flow cytometry. Our results indicated that abemaciclib and sitravatinib synergistically cooperated to reverse some characteristics of the immunosuppressive TME. Although there were no statistically significant changes in the frequencies of total, CD4+, or CD8+ T cells between treatment groups, the PD-1+ CD8 T-cell subset, associated with T-cell exhaustion, was significantly decreased in dual-treated mice (Figure 6b). PD-1+ CD4 T cells showed a similar trend but did not reach statistical significance. In addition, there was a decrease in the frequency of CD11b+ F4/80+ tumor-associated macrophages across all treatments, consistent with reduced tumor growth. Sitravatinib treatment significantly reduced the frequency of total CD11c+ dendritic cells, although there were no significant changes in the CD11b- and CD11b+ subsets. Moreover, the combined treatment significantly increased the frequency of CD3-CD49b+ natural killer cells, a key population involved in tumor control (Figure 6b and Supplementary Figure S8). Overall, these results suggest that the combination of abemaciclib and sitravatinib modifies the immune landscape of 4T1 tumors to favor an anti-tumor response.

## 4. Discussion

TNBC is associated with aggressive phenotype, treatment resistance, and high mortality, so developing an easily translatable therapeutic approach against it is a crucial goal. Prior studies aiming to find therapeutic leverage against TNBC have significantly contributed to our understanding of the biology of TNBC. Despite this, there has not been significant success in developing therapies that can be easily translated to the clinic.

CDK4/6 inhibitors have been successfully used against early or metastatic ER+/HER2− breast cancers [14,15,16,17]. Despite high levels of CDK6 and also CDK4 expression in TNBC, CDK4/6 inhibitors are not effective against this subtype due to the emergence of resistance. While changes in the cell cycle regulators, including the amplification of cyclin D, activation of CDK, and loss of p21^CIP1^ or p27^KIP1^, may contribute to tumor adaptation to CDK4/6 inhibition, the main resistance is mediated by *RB1* inactivation [60,61]. Since our aim is to develop a more effective combination therapy to overcome resistance to CDK4/6 inhibition, we decided to use the cell lines that are already resistant to CDK4/6 inhibition. For this reason, we picked the cell lines that do not express *RB1*. Several recent studies suggest inverse correlations between the RB pathway and mTOR activity [62,63]. With our previous study, we also demonstrated the activation of the mTOR pathway in glioblastoma as a resistance mechanism to CDK4/6 inhibition [40]. We initially showed that CDK4/6 inhibition significantly activates Akt and mTOR. This prompted us to search for a strategy to overcome the resistance to CDK4/6 inhibitors in the form of a combinatorial approach. Since these proteins are regulated by the upstream signaling from RTKs, we sought for the activation of RTKs in response to CDK4/6 inhibition.

RTKs play crucial roles in tumor growth, invasion, treatment resistance, and distant metastasis. We demonstrated that the treatment of TNBC lines with CDK4/6 inhibitors induces the activation of Met and TAM RTKs that subsequently drive therapeutic resistance. High Met activity is a known driver of carcinogenesis, tumor growth, and treatment resistance with shortened survival in breast cancer [64]. TAM RTKs have recently been implicated in tumor growth, metastasis, and therapeutic resistance in breast cancer [19,20,21,22,23]. Furthermore, the TAM receptors also promote the creation of an immunosuppressive TME [24,25,26,27], thereby constituting an attractive therapeutic target. In preclinical studies, targeting TAM RTKs has been successfully tested in combination with immune checkpoint inhibitors and chemotherapy in breast cancer [65]. Additionally, the pan-TAM inhibitor BMS-777607 was shown to enhance the efficacy of anti-PD-1 therapy in a murine TNBC model [66]. However, there has not been any progress in the clinical translation of these studies.

The crosstalk among RTKs is a dynamic process, enabling cancer cells to develop resistance to the treatment [67,68]. We showed that the Met and Axl pathways transactivate each other in TNBC to potentiate downstream signaling and promote CDK4/6 inhibitor resistance. Therefore, using a broad spectrum RTK inhibitor targeting the hyperactivated Met/TAM RTKs would be a more effective strategy to overcome resistance and increase efficacy. We showed that a clinically viable combination therapy, sitravatinib and abemaciclib, has significant efficacy against TNBC in vitro and in vivo. We showed that the combination therapy is synergistic, detected with two synergy calculation methods. While CDK4/6 inhibitors are cytostatic agents, we demonstrated that the combined treatment significantly induces apoptosis. We further verified our findings using a second agent, merestinib. Similar to sitravatinib, merestinib is a potent inhibitor of Met and TAM RTKs. With prolonged HER2 inhibitor treatment, HER2+ breast cancers suppress HER2 expression, so physiologically acquire a TNBC-like phenotype. Our initial results suggest that the combination therapy can also be effectively used against drug-resistant HER2+ breast cancer, another crucial unmet therapeutic challenge in breast cancer. Our findings suggest that employing RTK profiling can be an effective strategy to identify resistance mechanisms and help develop more effective combination therapies accordingly. In line with this, using a broad spectrum RTK inhibitor targeting multiple activated RTKs would likely yield better therapeutic outcomes.

Since TNBC is characterized by an immunosuppressive TME, it is likely that higher expression levels of the TAM receptors in TNBC vs. HER2+ breast cancer contribute to the creation of an immunosuppressive TME. Besides breast cancer cells, the TAM receptors are highly expressed in immune cells, including macrophages and NK cells. The activation of MerTK and Axl suppresses cytokine release and the inflammatory response [27]. The TAM receptors also negatively regulate natural killer cells and were shown to promote tumor metastases through suppressing their activities [26]. Consistent with these findings, we showed with immune profiling that the combined treatment significantly reversed the immunosuppressive TME, as evidenced by decreased CD8+ PD1 T cells and macrophages with increased NK cells (Figure 6b). The expression of PD1 on CD8+ T cells is a crucial marker of resistance to anti-PD1 therapy, and macrophages promote tumor growth and treatment resistance through driving immunosuppression in the TME [69,70]. Supporting our findings, sitravatinib has been successfully tested in several clinical trials to boost immune response against different solid tumors [51,52,53,54,55].

Given the translational nature of our study, it contains certain limitations. While we characterized the immune changes induced by the combination therapy, it is not clear whether any specific immune cells are predominantly responsible for the observed tumoricidal effect. For example, NK cells play a crucial role in the immune response against tumors, but their function can be regulated by various factors within the TME. Furthermore, macrophages exhibit a dual role, with the ability to both promote and inhibit tumor progression. Despite these limitations, our work offers a novel combination therapy that has a high potential to improve the clinical outcome. Given the recent clinical trials with sitravatinib, the combined treatment can be easily tested in the clinic against TNBC and potentially the drug-resistant HER2+ breast cancer.

## 5. Conclusions

It is now evident that monotherapies in general are not sufficient to provide sustained responses, necessitating the use of combination therapies. Following the findings that CDK4/6 inhibition additionally stimulates the immune response in breast cancer [28], several treatment strategies combining CDK4/6 and immune checkpoint inhibitors have been explored to boost immune response [71]. While this approach seems to be feasible, given the cytostatic nature of CDK4/6 inhibitors, it may fail to provide a durable response. Our findings suggest that the combination of sitravatinib with CDK4/6 inhibition may offer advantages over pure immunotherapeutic approaches. While sitravatinib treatment enhances the immune response in cooperation with abemaciclib, it also sensitizes the cancer cells to CDK4/6 inhibition by suppressing the RTKs—synergism through two complementary mechanisms.

## Data Availability

All data supporting the findings of this study are available from the corresponding author on reasonable request.

## Supplementary Materials

The following supporting information can be downloaded at https://www.mdpi.com/article/10.3390/cancers16122253/s1, File S1: The original western blot figures; Figure S1: Receptor Tyrosine Kinase pathway is activated upon CDK4/6 inhibition; Figure S2: CDK6 expression significantly correlates with the expression of TAM/Met; Figure S3: The combination of merestinib with abemaciclib or palbociclib is highly toxic against TNBC cells; Figure S4: Lapatinib-resistant HER2+ cell lines became more sensitive to the combination of merestinib with abemaciclib or palbociclib; Figure S5: The combined treatment significantly reduced tumor weights and sizes; Figure S6: Results from RNAseq differential expression analysis; Figure S7: Enrichment analysis for GO suggests the activation of biological processes associated with the endo-lysosomal pathway; Figure S8: Gating strategy for the phenotyping.
