# Supplementary material for: Targeting Tyro3, Axl, and MerTK Receptor Tyrosine Kinases Significantly Sensitizes Triple-Negative Breast Cancer to CDK4/6 Inhibition
Source: Cancers (Basel). 2024 Jun 18;16(12):2253. doi: 10.3390/cancers16122253 (PMC11202171; doi:10.3390/cancers16122253)
Supplement: Supplementary file 1 [file cancers-16-02253-s001.zip › cancers-3027832-supplementary figures.pdf]

Supplementary Data Figures and Legends

a

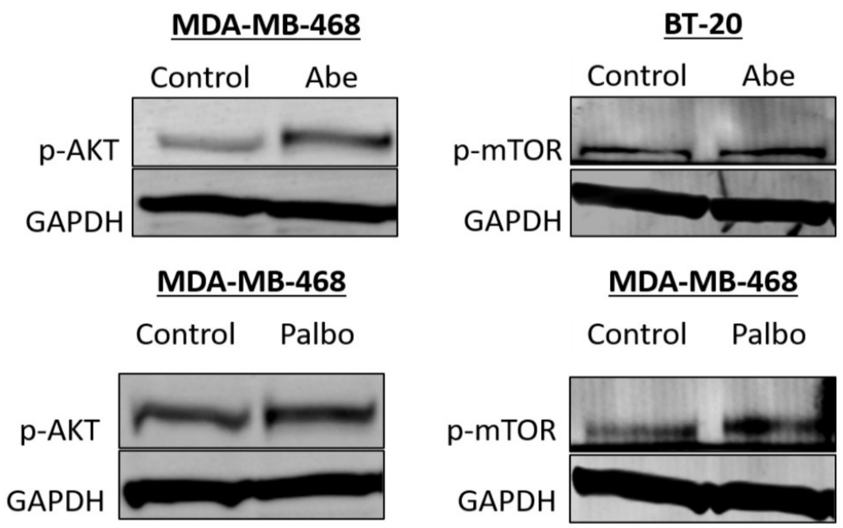

b

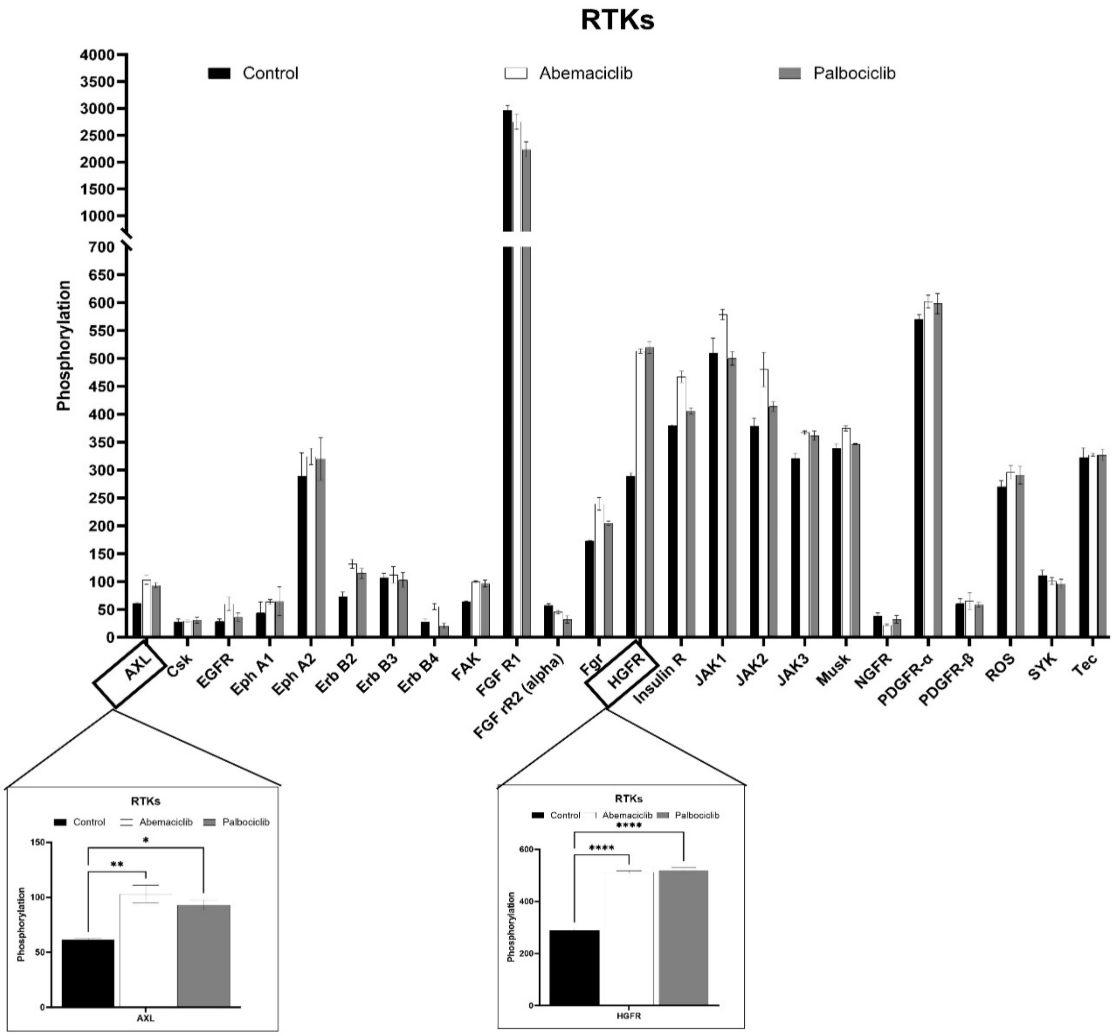

Supplementary Figure S 1 : Receptor Tyrosine Kinase pathway is activated upon CDK4/6 inhibition. a, Immunoblot was performed on cell lines treated for 24h with Abe (2 uM) or Palbo (5 uM). Protein levels were determined for phospho-AKT, phosho-mTOR, and GAPDH. **b,** The Phospho-RTK array developed using lysates from MDA-MB-231 cells treated with Abe (2 uM) or Palbo (5 uM, 24 h) for 24h (* $p < 0.05$, ** $p < 0.01$, **** $p < 0.0001$; one-way ANOVA with Dunnett's multiple comparisons test analysis). Abe: abemaciclib, Palbo: Palbociclib.

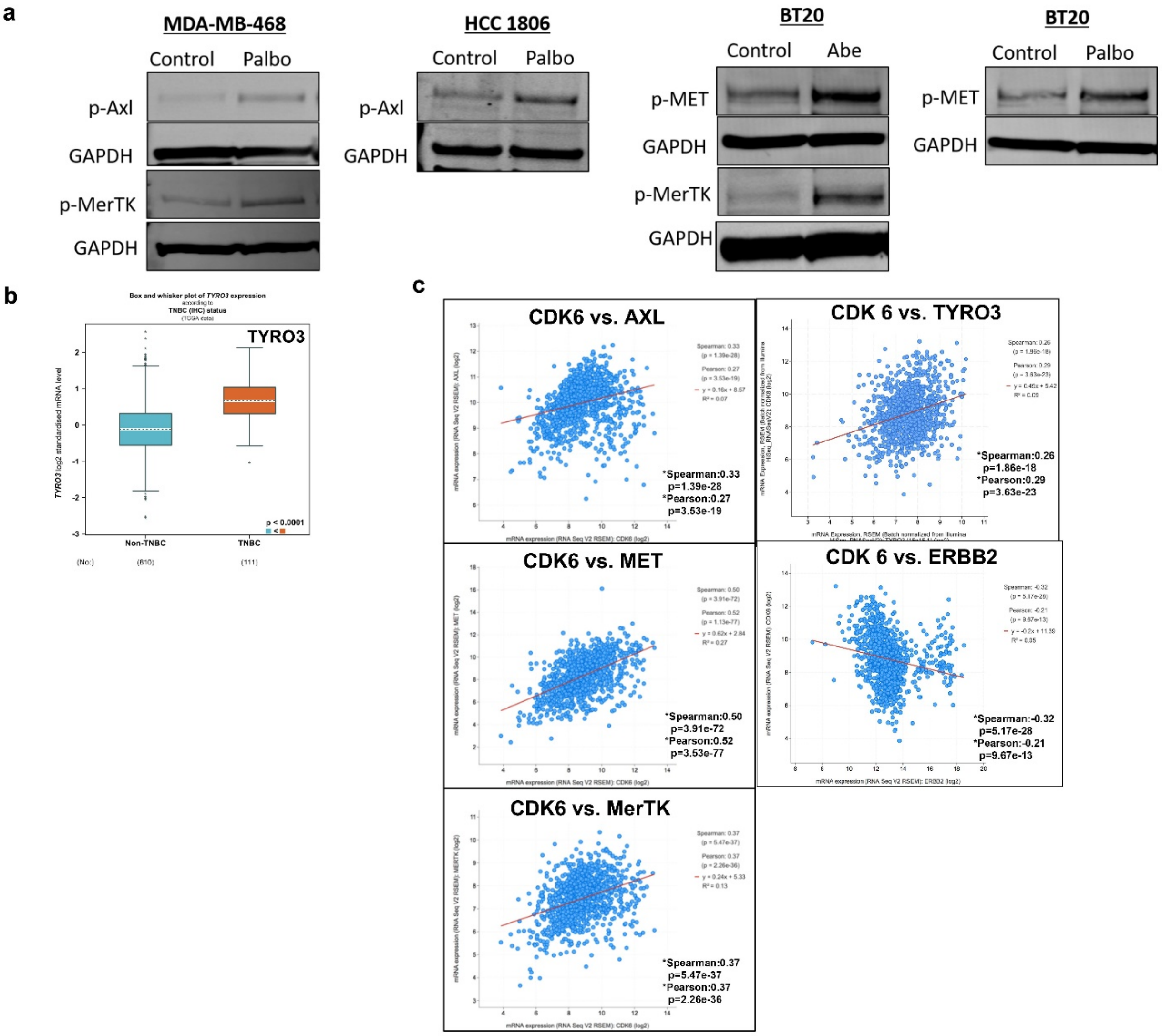

Supplementary Figure S2: CDK6 expression significantly correlates with the expression of TAM/Met. a, Immunoblot was performed on cell lines treated for 24h with Abe (2 uM) and Palbo (5 uM). Protein levels were determined for phospho-AXL, phosho-MET, and phosho-MerTK. **b,** Comparison of Tyro3 gene expression levels in TNBC vs. non-TNBC, based on RNAseq data from breast cancer patients. **c,** TCGA data on breast cancer patients showing positive correlations between CDK6 and TAM/Met RTKs and inverse correlation between CDK6 and ERBB2 (Her2). Data were taken from TCGA database for breast cancer patients (cBioPortal, PanCancer Atlas). Abe: abemaciclib, Palbo: palbociclib.

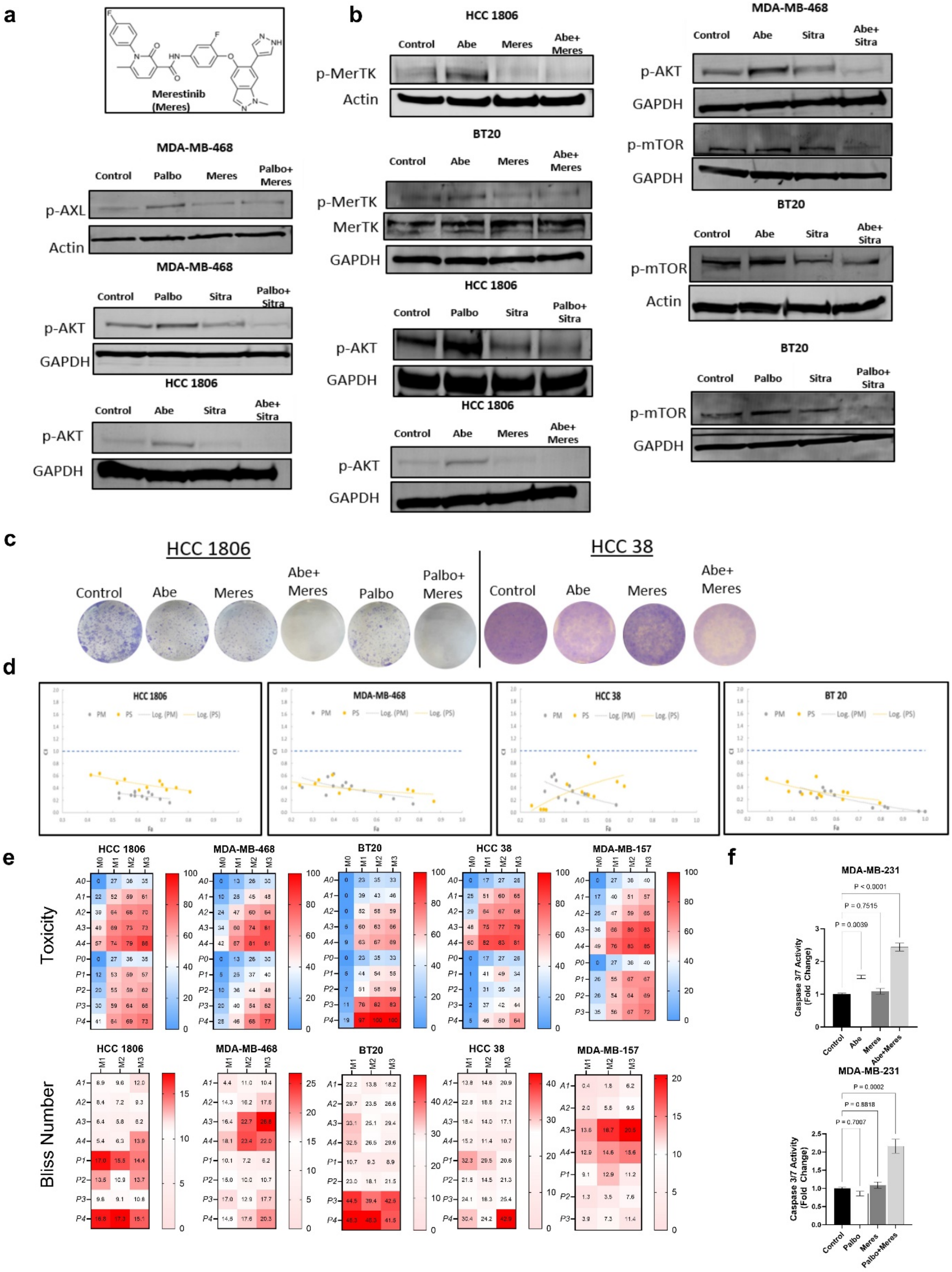
a
Merestinib (Meres)
MDA-MB-468
Control Palbo Meres Palbo+ Meres
p-AXL
Actin
MDA-MB-468
Control Palbo Sitra Palbo+ Sitra
p-AKT
GAPDH
HCC 1806
Control Abe Sitra Abe+ Sitra
p-AKT
GAPDH
b
HCC 1806
Control Abe Meres Abe+ Meres
p-MerTK
Actin
BT20
Control Abe Meres Abe+ Meres
p-MerTK
MerTK
GAPDH
HCC 1806
Control Palbo Sitra Palbo+ Sitra
p-AKT
GAPDH
HCC 1806
Control Abe Meres Abe+ Meres
p-AKT
GAPDH
MDA-MB-468
Control Abe Sitra Abe+ Sitra
p-AKT
GAPDH
p-mTOR
GAPDH
BT20
Control Abe Sitra Abe+ Sitra
p-mTOR
Actin
BT20
Control Palbo Sitra Palbo+ Sitra
p-mTOR
GAPDH
c
HCC 1806
Control Abe Meres Abe+ Meres Palbo Palbo+ Meres
HCC 38
Control Abe Meres Abe+ Meres
d
HCC 1806
MDA-MB-468
HCC 38
BT 20
e
Toxicity
HCC 1806
MDA-MB-468
BT20
HCC 38
MDA-MB-157
Bliss Number
HCC 1806
MDA-MB-468
BT20
HCC 38
MDA-MB-157
f
MDA-MB-231
P < 0.0001
P = 0.7515
P = 0.0039
Caspase 3/7 Activity (Fold Change)
Control Abe Meres Abe+Meres
MDA-MB-231
P = 0.0002
P = 0.8818
P = 0.7007
Caspase 3/7 Activity (Fold Change)
Control Palbo Meres Palbo+Meres

Supplementary Figure S3: The combination of merestinib with abemaciclib or palbociclib is highly toxic against TNBC cells. a, Chemical structure of merestinib (Meres). **b,** Immunoblot was performed on cell lines treated for 24h with Abe (2 uM), Palbo (5 uM), Sitra (2 uM), and Meres (6 uM). Protein levels were determined for phospho-AXL, phosho-MERTK, phosho-AKT, and phosho-mTOR. **c,** The clonogenic assay showing that the combination of Abe or Palbo with Meres significantly decreased the colony formation capacity of TNBC cells. Representative images of stained colonies. **d,** Combination Index (CI) values for the combinations of sitravatinib or merestinib with CDK4/6 inhibitor palbociclib using different doses. Circles represent experimentally determined CI values using the Chou Talalay method. The colors (yellow and gray) represent the fixed ratio mixtures. **e,** Overview of the toxicity and synergy scores of the drug combinations. The heatmaps show the level of Toxicity and Bliss number for the cell lines tested. Average values of Toxicity or Bliss Number for cells treated with merestinib (M) at varying doses (M0= No Drug, M1= 1 uM, M2=2 uM and M3=3 uM) in combination with either abemaciclib (A) at varying doses (A0= No Drug, A1= 1 uM, A2=2 uM and A3=3 uM, A4= 4 uM) or palbociclib (P) at varying doses (P0= No Drug, P1= 1 uM, P2=2 uM and P3=3 uM, P4= 4 uM). **f,** Shown is the caspase-3/7 activity measured upon 24h of drug treatments. The data are presented as mean ± SEM from three independent experiments, expressed as ratios to untreated control values, with associated p values as indicated (one-way ANOVA with Dunnett's multiple comparisons test analysis). Abe: abemaciclib, Palbo: palbociclib, Sitra: sitravatinib.

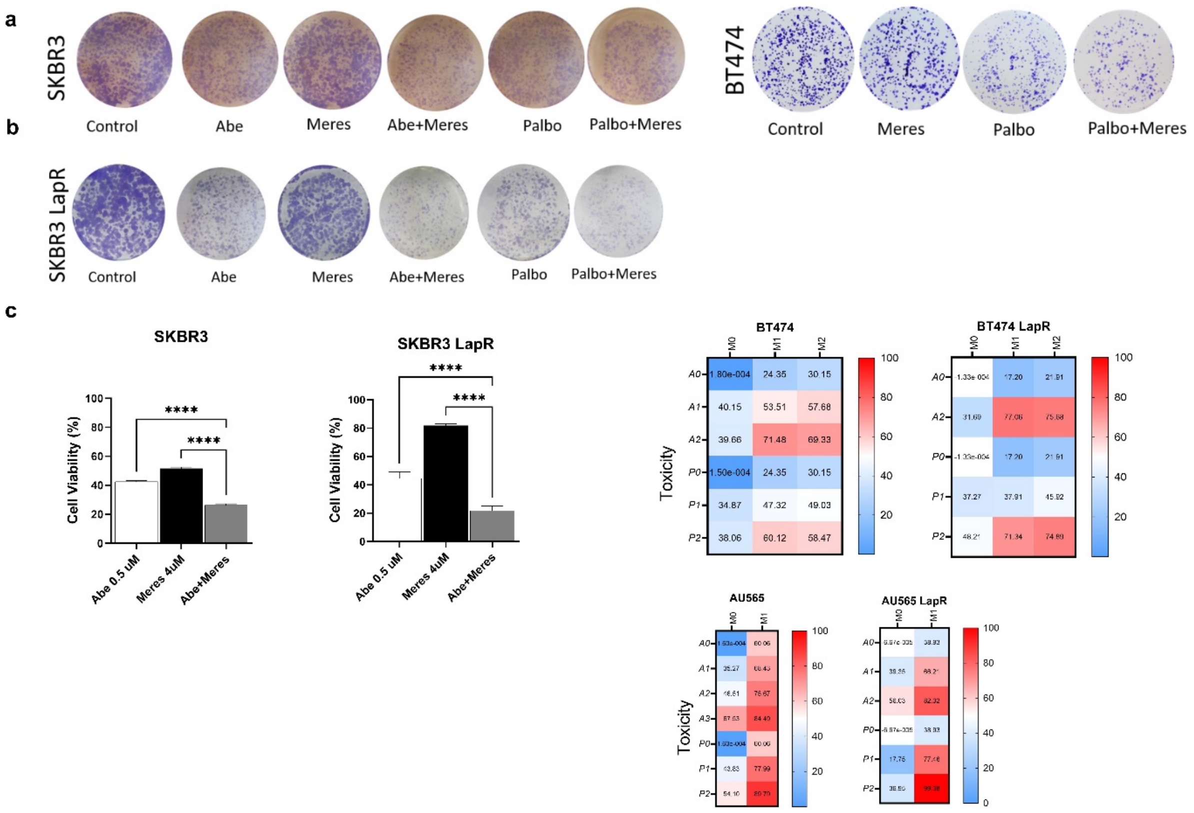

Supplementary Figure S4: Lapatinib-resistant HER2+ cell lines became more sensitive to the combination of merestinib with abemaciclib or palbociclib. a, The clonogenic assay showing that the combination of Abe or Palbo and merestinib (Meres) had only modest effect on the HER2+ cell lines SKBR3 and BT474. **b,** The clonogenic assay showing that SKBR3-LapR cells became highly sensitive to the combination of Abe or Palbo with Meres. **c,** Cell viability showing increased sensitivity of SKBR3 LapR cells to the combination of abemaciclib or palbociclib with merestinib compared with the parental SKBR3 cells. Each bar represents as mean ± SEM from three independent experiments, with associated p (**** $p < 0.0001$; one-way ANOVA with post-hoc Tukey analysis; either compared with untreated or the single agent groups). Overview of the toxicity of the drug combinations for HER2+ and LapR HER2 cell lines (right panel). The heatmaps show the level of Toxicity for the cell lines tested. Average values of Toxicity for cells treated with Meres (M) at varying doses (M0= No Drug, M1=4 uM for AU565 and AU565 LapR; and M1= 5 uM, M2= 6 uM for BT474 and BT474 LapR) in combination with either Abe (A) (A0= No Drug, A1= 2 uM) or Palbo (P0= No Drug, P1 =1 uM and P2= 2 uM for AU565 and AU565 LapR; and P1= 5 uM, P2=6 uM for BR474 and BT474 LapR). Abe: abemaciclib, Palbo: palbociclib, Meres: merestinib.

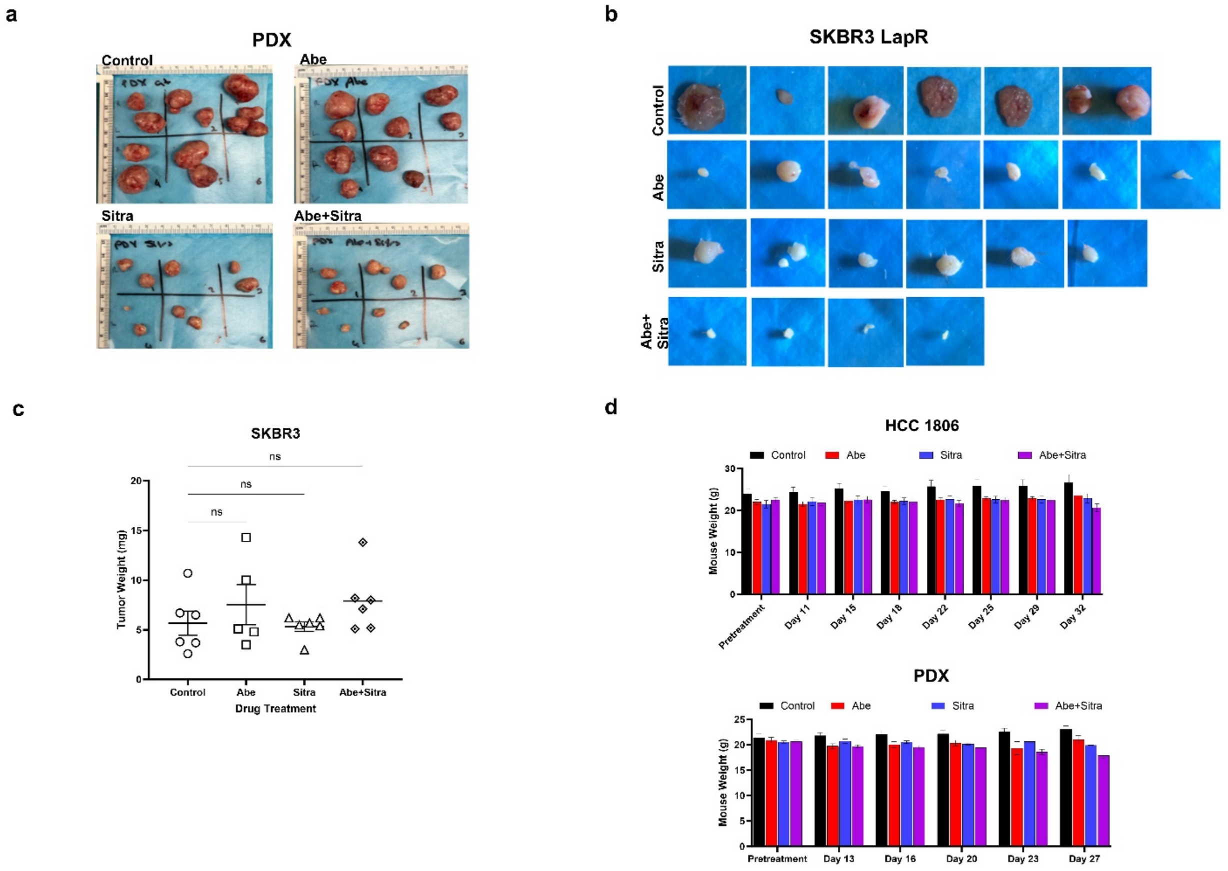

Supplementary Figure S5: The combined treatment significantly reduced tumor weights and sizes. a,b Shown are tumor images of the treatment groups for the PDX and SKBR3-LapR. **c**, Tumor weight comparison showing no significant difference across the treatment groups with the HER2+ cell line SKBR3 (one-way ANOVA on ranks with Dunn's multiple comparison test analysis). **d,** The average body weight of mice in each group remained similar over the course of the treatment.

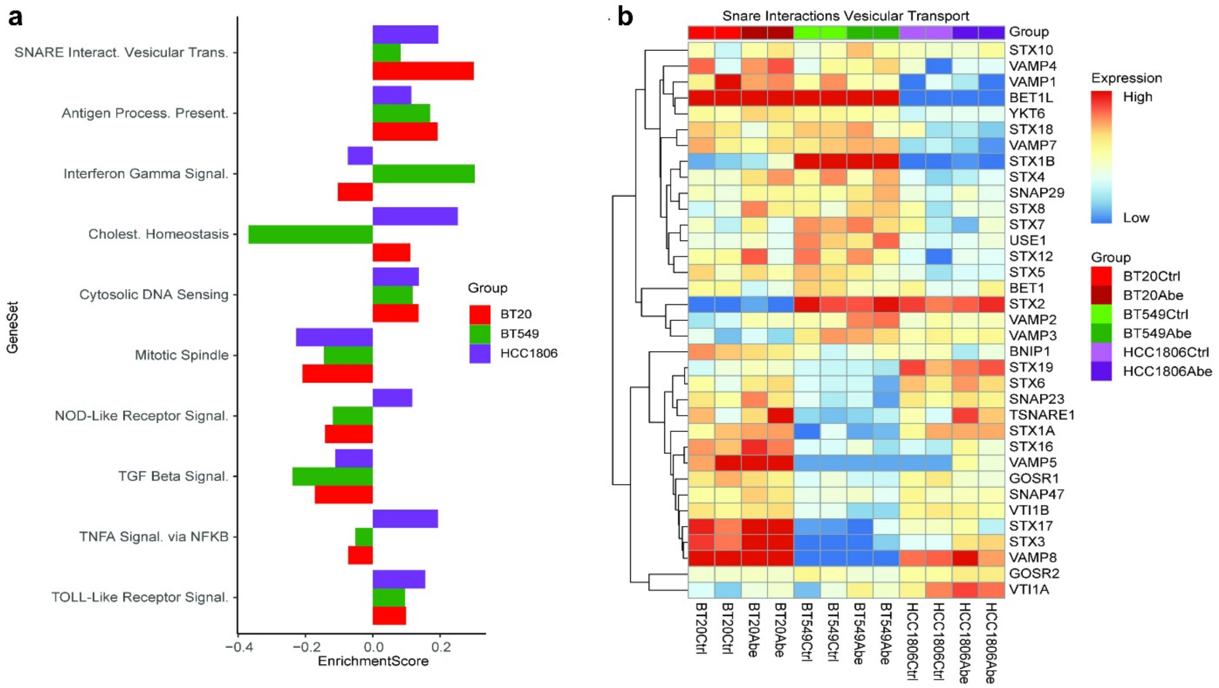

Supplementary Figure S6: Results from RNAseq differential expression analysis. a, Barplot display of enrichment scores from a gene set enrichment analysis (GSEA) pre-ranked analysis for select Hallmark and KEGG gene sets. **b,** Gene expression heatmap for genes associated with the SNARE complex. Columns are grouped by cell line and treatment. RNAseq data were obtained from GSE99116.

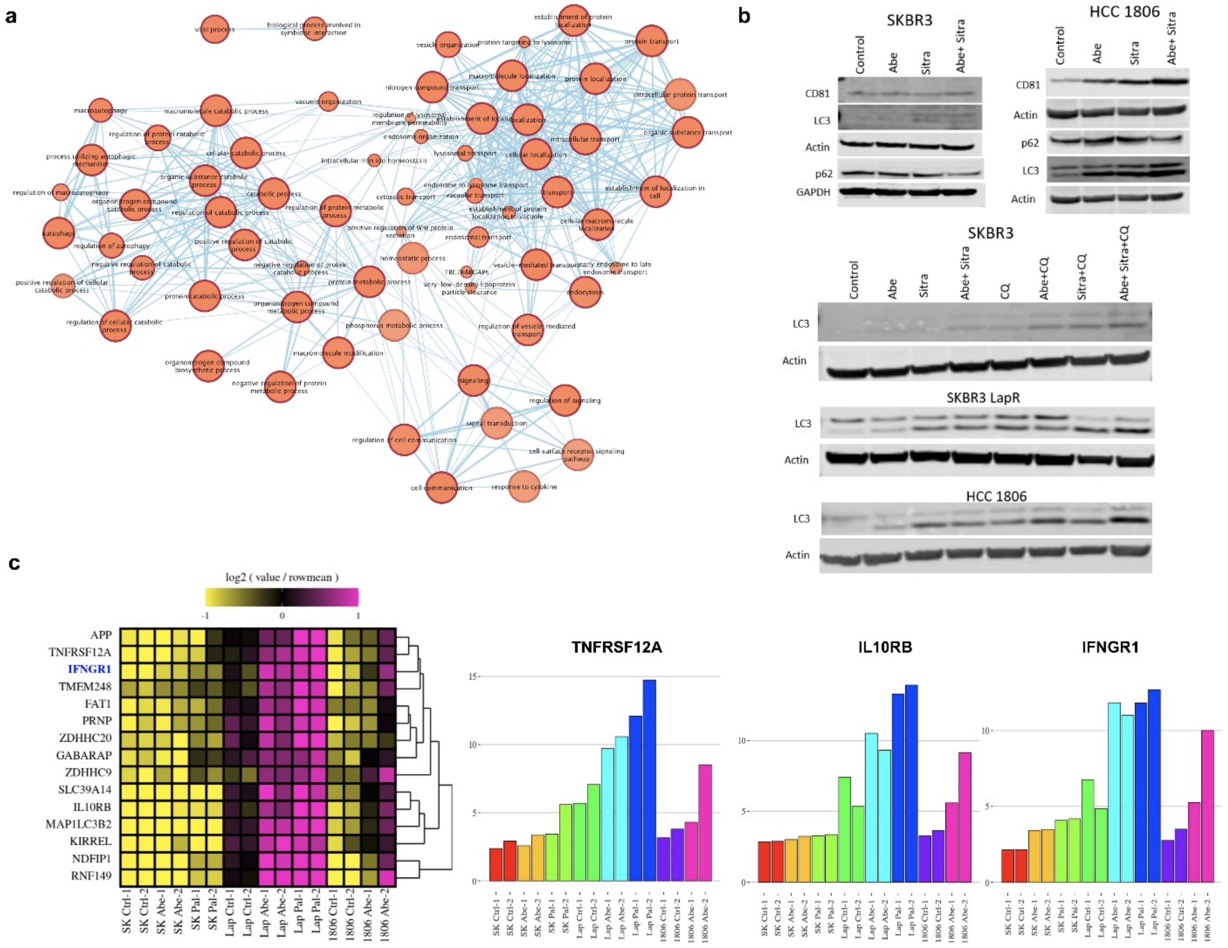

Supplementary Figure S7: Enrichment analysis for GO suggests the activation of biological processes associated with the endo-lysosomal pathway. a, Map visualization of the enriched GO-Biological Processes activated in HCC1806 and SKBR3-LapR. GO analysis was performed on g:Profiler and visualized using Cytoscape. **b,** Immunoblot was performed on cell lines pretreated with chloroquine (CQ) (10 uM) followed by 24h treatment with Abe (2 uM), Sitra (2.5 uM), or the combination of Abe and Sitra. Protein levels were determined for CD81, LC3, and p62. **c,** Heatmap of the top upregulated proteins in HCC1806 and SKBR3-LapR vs. SKBR3. Barplots showing the expression levels of proteins across the cell lines before and after treatments with abemaciclib and palbociclib.

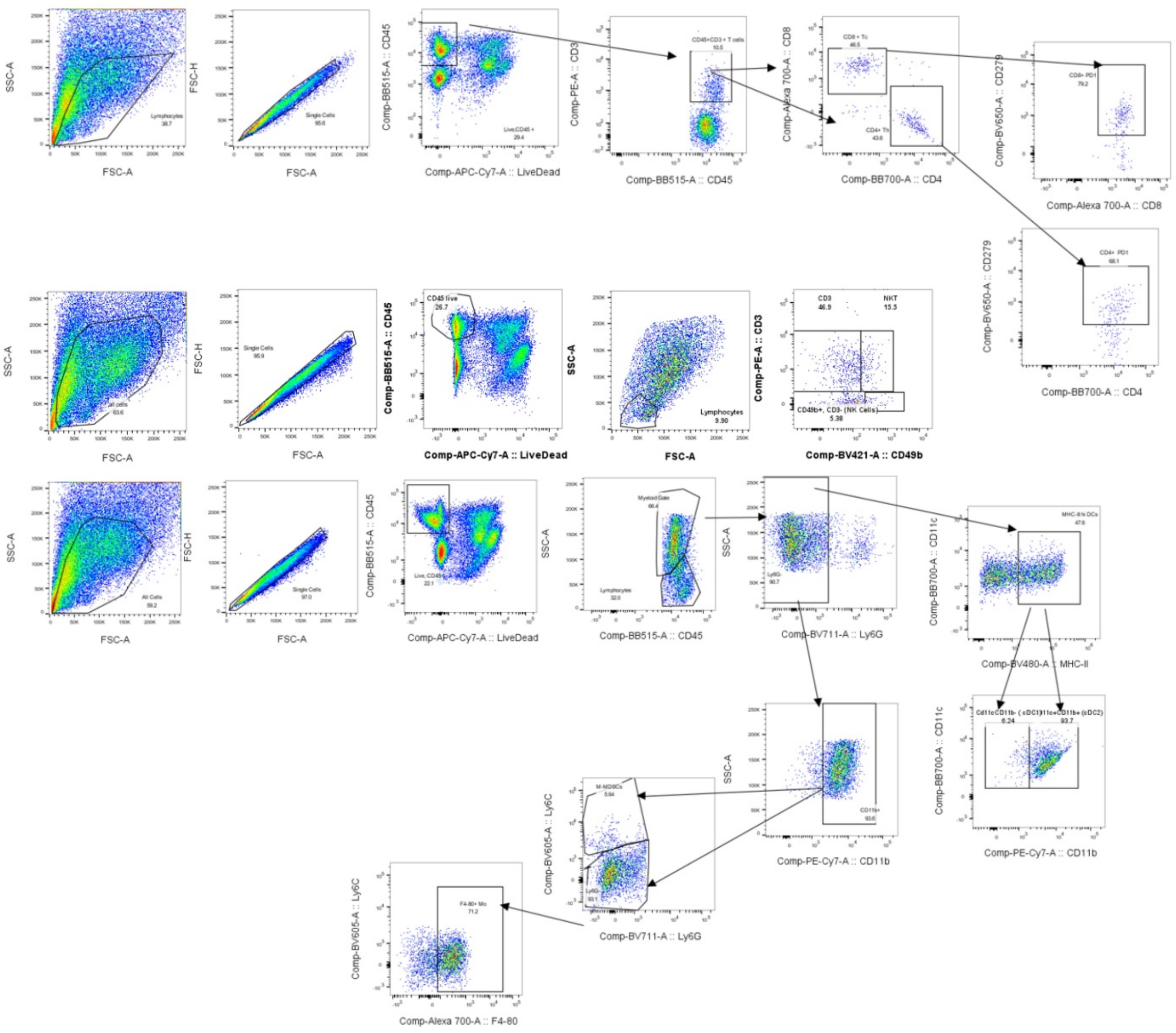

Supplementary Figure S8: Gating strategy for the phenotyping. Gating strategy for the phenotyping of the cell subpopulations from the excised 4T1 tumors.
